# MicroRNA-663 facilitates the growth, migration and invasion of ovarian cancer cell by inhibiting TUSC2

**DOI:** 10.1186/s40659-019-0219-6

**Published:** 2019-04-03

**Authors:** Hui Hui Xie, Wen Ting Huan, Jiang Qiong Han, Wei Ru Ren, Li Hua Yang

**Affiliations:** 1 Obstetrics and Gynecology, Xuzhou Central Hospital, Xuzhou, Jiangsu China; 2 Department of Integrated Traditional Chinese and Western Medicine, The Third Affiliated Hospital of Kunming Medical University (The Tumor Hospital of Yunnan Province), Kunming, Yunnan China; 3 Gynaecology Ward of Maternal and Child Health Hospital, Zaozhuang, Shandong China; 4 Department of Gynaecology, the 2nd Affiliated Hospital of Kunming Medical University, Kunming, Yunnan China

**Keywords:** MiR-663, Ovarian cancer, TUSC2, Growth, Migration, Invasion

## Abstract

**Background:**

MicroRNAs (miRNAs) have emerged as the critical modulators of the tumorigenesis and tumor progression.

**Methods:**

The levels of miR-663 in ovarian cancer cell lines and clinical tissues were detected using qRT-PCR assays. The Transwell invasion and wound healing assay were conducted to assess the roles of miR-663 in the migration and invasion of ovarian cancer cell in vitro. Rescue assays were carried out to confirm the contribution of tumor suppressor candidate 2 (TUSC2) in the aggressiveness of cancer cell which was regulated by miR-663.

**Results:**

The levels of miR-663 were up-regulated in ovarian cancer tissues in comparison with the corresponding normal tissues. Up-regulation of miR-663 increased the proliferation, colony formation, migration and invasion of ovarian cancer SKOV3 cell. Additional, over-expression of miR-663 increased the tumor growth of SKOV3 in xenograft model. Bioinformatics analysis and luciferase reporter assay identified that miR-663 decreased the level of TUSC2 via binding to the 3′-UTR of TUSC2 gene. Finally, the expression of TUSC2 was inversely associated with the level of miR-663 in ovarian carcinoma tissue and over-expression of TUSC2 inhibited the migration and invasion abilities of SKOV3 that was promoted by miR-663.

**Conclusion:**

Altogether, these results indicate that miR-663 acts as a potential tumor-promoting miRNA through targeting TUSC2 in ovarian cancer.

**Electronic supplementary material:**

The online version of this article (10.1186/s40659-019-0219-6) contains supplementary material, which is available to authorized users.

## Background

Ovarian carcinoma is one of the commonly gynecological malignancies with unoptimistic clinical outcomes [1, 2]. Although the remarkably improvement in the treatment options for ovarian cancer in the last few years, the 5-year survival rate of this disease is not optimistic, mainly due to advanced stage at diagnosis and the recurrence and metastasis [3, 4]. Ovarian cancer often metastasizes throughout the peritoneal cavity, to the omentum, and even to the parenchyma of the liver or lung. Many investigations have indicated that the metastasis of ovarian cancer cell could be divided into several steps: (I) cell detachment, survival, and resistance of anoikis; (II) evasion of immunological surveillance; (III) epithelial-mesenchymal transition; (IV) spheroid formation; (V) ascites formation; and (VI) peritoneal implantation [5, 6]. Because > 70% of ovarian cancer cases are diagnosed at a late stage, when cancer cells are actively metastasizing, understanding ovarian cancer pathogenesis and the mechanism of its metastasis is crucial for the management of this deadly, highly metastatic disease [7]. Hence, elaborating the novel-innovative mechanism of ovarian cancer metastasis are crucial to exploitation new therapeutic strategy [8].

MicroRNAs (miRNAs), which belong to a kind of conserved noncoding RNAs, regulate the expressions of target proteins in posttranscriptional dependent manner through binding to 3′-untranslated region (3′-UTR) of genes [9]. The roles of alternated miRNAs in diverse biological processes, including tumor cell growth, cellular apoptosis and chemotherapy resistance have been exactly demonstrated [10]. MiRNAs function as tumor suppressor or oncogenic miRNA in the tumorigenesis and cancer progression based on the functions of their target genes [11]. Previous studies have shown the role of miR-663 in many important pathological processes, including autoimmune diseases, infection, and inflammatory reaction [12, 13]. However, its function in tumor progression is contradictory. Although it acts as a cancer promoter in breast cancer and nasopharyngeal carcinoma, miR-663 may also be shown as a potential tumor suppressor in glioblastoma and gastric cancer [14, 15]. However, to our knowledge, no functional evidence of miR663 in ovarian carcinoma has been documented.

Tumor suppressor candidate 2 (TUSC2), which is also known as FUS1 in chromosome 3p21.3 region, has been identified to function as a anti-oncogene [16]. Allele losses and genetic alterations of TUSC2 are found in multitudinous cancer types, including lung carcinoma and breast cancer [17]. For example, TUSC2 serves as a tumor-suppressor via up-regulating the level of miR-197 in glioblastoma [18]. Currently, TUSC2 is down-regulated in nasopharyngeal carcinoma (NPC) genes and relates to cellular apoptosis in the chromosome deletion regions. Nevertheless, the expression of TUSC2 gene in ovarian cancer remains not well investigated.

In this study, we demonstrated that miR-663 was up-regulated in ovarian cancer. Furthermore, over-expression of miR-663 promoted the growth, migration and invasion of ovarian cancer cell. In addition, TUSC2 was identified as the target gene of miR-663 and its expression was negatively regulated by miR-663. Finally, we found that miR-663 promoted the aggressiveness of ovarian carcinoma cellvia targeting TUSC2.

## Methods

### Cell lines and tissue samples

Ovarian carcinoma cells (SKOV3, HO-8910, OVCAR, A2780) and the ovarian surface epithelial cell line, HOSEpiC, were purchased from Jennio Biotech Co., Ltd (GuangZhou, Guangdong, China). Cells were cultured with 1640 or DMEM (Invitrogen, Carlsbad, CA, USA) containing 10% FBS and 1% penicillin/streptomycin. Twenty-three pairs of ovarian cancer tissues and adjacent normal tissues were obtained from Gynaecology Ward of Maternal and Child Health Hospital (Zaozhuang, Shandong Province, China) and Xuzhou Central Hospital. Prior patients’ consent and ethics approval were obtained from the Gynaecology Ward of Maternal and Child Health Hospital and Xuzhou Central Hospital. All tissues were immediately frozen in liquid nitrogen for future tests.

### Transfection

SKOV3 cell line was selected for investigating the role of miR-663 in ovarian carcinoma owing to the level of miR-663 was highest in SKOV3. Cells at confluency of 40–50% were transfected with miRNAs (5 μg/well for 6 well culture plates) using Lipofectamine 2000 reagent (Invitrogen; Carlsbad, Calif, USA). The miR-663 mimic and a nonspecific miR control (miR-NC) were purchased from GenePharma (Shanghai, China). The expression construct of TUSC2 was produced by subcloning PCR-amplied full-length human TUSC2 cDNA into the pCDNA3.1 plasmid (Invitrogen, Carlsbad CA, USA). For cotransfection, SKOV3 cells were transiently transfected with plasmids (2 μg/well for 6 well culture plates) containing the TUSC2 and miR-663 mimic (5 μg/well for 6 well culture plates) using Lipofectamine 2000 reagent (Invitrogen, Carlsbad, Calif, USA) according to the manufacturer’s recommendations.

### Quantitative real-time PCR

RNA was extracted using TRIzol reagent (Invitrogen CA, USA). RNA (1 µg) was reversely transcribed into cDNA using the PrimeScript RT reagent kit (TakaraBio, Tokyo, Japan). qRT-PCR was conducted using SYBR Premix Ex Taq™ kit (TakaraBio) on the Applied Biosystems 7900 Sequence Detection system (Applied Biosystems). For detecting miR-663, the Mir-VanaTM MiRNA Isolation Kit (Ambion, USA) was used to isolate total RNA from cell lines and patient samples following the manufacturer’s instructions. MiR-663 was detected using Platinum Taq DNA Polymerase (Invitrogen) on the Applied Biosystems 7900 Sequence Detection system (Applied Biosystems). The primers were as follows: TUSC2 (forward primer: 5′-GGAGACAATCGTCACCAAGAAC-3′; reserve primer: 5′-TCACACCTCATAGAGGATCACAG-3′), U6 (forward primer: 5′-AAAGCAAATCATCGGACGACC-3′; reverse primer: 5′-GTACAACACATTGTTTCCTCGGA-3′), TGFβ1 (forward primer: 5′-GTAGCTCTGATGAGTGCAATGAC-3′; reverse primer: 5′-CAGATATGGCAACTCCCAGTG-3′); eEF1A2 (forward primer: 5′-GAAGACCCACATCAACATCGT-3′; reverse primer: 5′-CTCCGCATTTGTAGATGAGGTG-3′); HRAS (forward primer: 5′-TGCTTCAGTTTGAACTACCCTG-3′; reverse primer: 5′-GCCCAGTGCTGATAGCCAG-3′); PIK3CD (forward primer: 5′-AAGGAGGAGAATCAGAGCGTT-3′; reverse primer: 5′-GAAGAGCGGCTCATACTGGG-3′); CXCR4 (forward primer: 5′-ACTACACCGAGGAAATGGGCT-3′; reverse primer: 5′-CCCACAATGCCAGTTAAGAAGA-3′); GAPDH (forward primer: 5′-TGTGGGCATCAATGGATTTGG-3′; reverse primer: 5′-ACACCATGTATTCCGGGTCAAT-3′). U6 forward: CTCGCTTCGGCAGCACA, U6 reverse: AACGCTTCACGAATTTGCGT. U6 and GAPDH were the internal controls. The reaction condition was 95 °C for 5 min, followed by 40 cycles of denaturation at 95 °C for 15 s and annealing/elongation step at 60 °C for 30 s. The comparative cycle threshold (Ct) method was selected to detect the level by calculating the 2^(−∆∆Ct)^.

### Cells proliferation analysis

SKOV3 cell growth in vitro was detected using CCK-8 (Beyotime, Nanjing, Jiangsu, China) method. 2 × 10^3^ SKVO3 cells were cultured in 96-well plate for continued 24 h, 48 h, 72 h or 96 h, respectively. After each time node, each well was added CCK-8 solution (10 μL) and after 1 h, the OD was detected at 450 nm.

### Clone formation analysis

500 cells/well miR-663 or miR-NC transfected ovarian carcinoma SKOV3 cells were plated into 6-well plate, and cells were cultured for 2 weeks. Cell colonies were stained using 1% crystal violet and the number of cell colonies was counted.

### Cell migration assay

1 × 10^5^/well transfected cells were grown in six-well plates and formed confluence. The cell confluence was swiped using a 100 μL tip to generate a gap. The SKOV3 cells wound were observed at 0 and 24 h using a ZEISS invert microscope (CarlZeiss, Germany) [19].

### Cell invasion analysis

Cell invasiveness was detected using the Transwell invasion assay. 1 × 10^5^ SKOV3 cells were plated into Matrigel coated upper chamber of Transwell. Medium containing 20% FBS was plated into lower chamber of Transwell. After 24 h, invaded SKOV3 cells were fixed with 4% methyl aldehyde and stained using 1% crystal violet. The number of invaded cell was counted in five randomly fields [20].

### Luciferase reporter analysis

3′-UTR-TUSC2 gene containing the binding site of miR-663 was PCR amplified and then sub-cloned into the psiCHECK2 (Promega, Madison, WI, USA). Mutant type 3′-UTR-TUSC2 gene was established using the Agilent Technologies QuikChange XL Site Directed Mutagenesis Kit and sub-cloned into the psiCHECK2. In luciferase reporter test, SKOV3 cells were co-transfected with miR-663 along with WT-UTR-TUSC2-3′ or MT-3′-UTR-TUSC2. The luciferase activity was measured in the luciferase reporter assay system (Promega).

### Immunoblotting

Cells were lysed using RIPA (Beyotime, Nanjing, Jiangsu, China) and total protein was quantitated using the BCA kit (Thermo, Rockford, USA). Equal amounts of cell lysates (25 μg) were loaded on 8% SDS-PAGE and transferred onto PVDF membranes.. The membranes were blocked with 5% nonfat dry milk in Tris-buffered saline with Tween 20 (TBST) for 1 h at 37 °C. The membrane was incubated with antibodies against TUSC2 (1:1000, Santa Cruz Biotechnology, Dallas, Texas, USA) and internal control GAPDH (1:1000, Bioworld Technology, Nanjing, Jiangsu, China), followed by incubating with the HRP-linked secondary antibody (1:10,000, Bioworld Technology, Nanjing, Jiangsu, China). The peroxidase activity was detected by ECL chemilumininescence detection kit (Pierce) and signal intensity of the protein bands were measured using the ChemiDoc XRS system (Bio-Rad, Hercules, CA, USA).

### Tumor xenograft model

Animal experiment was approved by the Animal Research Committee of Xuzhou Central Hospital. Tumor xenograft model was established using BALB/c nude mice by subcutaneously injecting 2 × 10^6^ miR-663 or miR-NC transfected SKOV3 cells. Tumor volume was monitored once every 3 days by assess the tumor width and length. The formula of tumor volume is (length × width^2^)/2. After 25 days, tumor mass were exfoliated and weighed.

### Statistical analysis

GraphPad Prism was used for statistical analysis. The data were presented as mean ± SD. Differences in the results of two groups were evaluated using either two-tailed Student’s *t* test or one-way ANOVA followed by post hoc Dunnett’s test. The differences with *P *< 0.05 were considered statistically significant. The connection between TUSC2 and miR-663 was tested by Spearman’s correlation analysis.

## Results

### MiR-663 is up-regulated in ovarian cancer

**Fig. 1:**
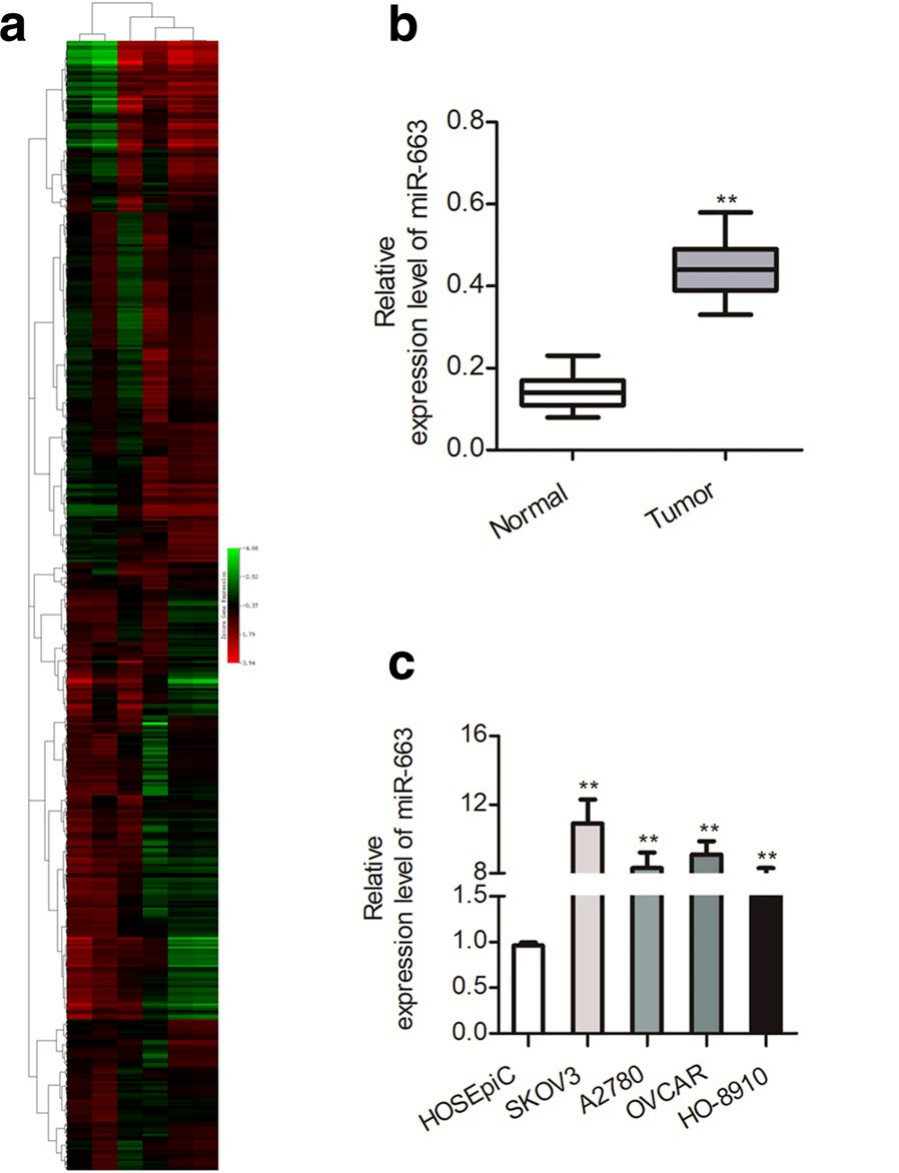
The level of miR-663 is over-expressed in ovarian carcinoma. **a** Microarray analysis of miRNA expression in ovarian cancer tissues from normal control tissues. **b** The level of miR-663 in 23 paired ovarian cancer samples and matched normal tissues was assessed by qRT-PCR. **P < 0.01, compared to normal. **c** qRT-PCR assayed of miR-663 in ovarian carcinoma cells (OVCAR, SKOV3, A2780, and HO-8910) and HOSEpiC. N = 3, **P < 0.01, compared with HOSEpiC cells

To investigate the dysregulation of miRNAs in cervical cancer, the GEO dataset, GSE83693 that containing primary ovarian cancer tissue and normal ovarian tissue was selected to explore the expression patterns of miRNAs. As shown in Fig. 1a, the heatmap that was generated using differential levels of miRNAs indicated that miR-663 was significantly up-regulated in ovarian cancer tissues. We then determined the levels of miR-663 in 23 pairs of ovarian cancer and corresponding non-cancer tissues using qRT-PCR method. As shown in Fig. 1b, miR-663 was significantly up-regulated in ovarian cancer tissues when compared with the matched normal tissues. Consistently, the level of miR-663 was higher in a panel of ovarian carcinoma cell lines (A2780, SKOV3, HO-8910 and OVCAR) than that in HOSEpiC (Fig. 1c). All these findings indicate that miR-663 is significantly up-regulated in both ovarian cancer samples and cell lines.

### Up-regulation of miR-663 promotes the proliferation of ovarian cancer cell

**Fig. 2:**
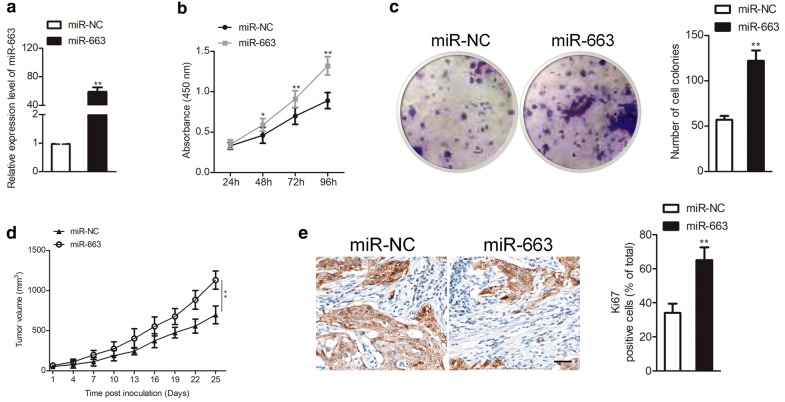
MiR-663 facilitates ovarian carcinoma SKOV3 cells growth in vitro and in vivo. **a** SKOV3 cells were transfected with either miR-663 or miR-NC, and the level of miR-663 was measured using qRT-PCR method. **b** The proliferation of miR-663 or miR-NC transfected SKOV3 cells was detected by CCK-8 assay. **c** Cells colonies in either miR-663 or miR-NC transfected SKOV3 cells. **d** Nude mice were subcutaneously inoculated with miR-NC or miR-663 transfected SKOV3 cells and xenograft tumor volumes were measured. **e** Representative image of Ki67 immunohistochemical staining in indicated xenograft tumors. N = 3, **P < 0.01 in comparison to miR-NC

To future explore the precise functions of miR-663 in ovarian carcinoma cell, SKOV3 cells were selected and transfected with miR-NC or miR-663 (Fig. 2a). CCK-8 experiment suggested that over-expression of miR-663 increased SKOV3 cell proliferation (Fig. 2b). Moreover, up-expression of miR-663 remarkably increased the colony formation of SKOV3 cell in vitro (Fig. 2c). To vitrify these observations, the miR-NC or miR-663 transfected SKOV3 cells were inoculated subcutaneously into nude mice. We found that the tumor growth was increased in mice that was inoculated with miR-663 transfected cells than that in the miR-NC group (Fig. 2d). Consistently, the Ki67 staining in tumor tissue that was formed by miR-663 transfected SKOV3 cell was remarkably increased than that in the tumor tissue that derived from miR-NC transfected SKOV3 cell (Fig. 2e). All results uncover that over-regulation of miR-663 promotes ovarian cancer growth in vitro and in vivo.

### MiR-663 promotes ovarian cancer cell migration and invasion

**Fig. 3:**
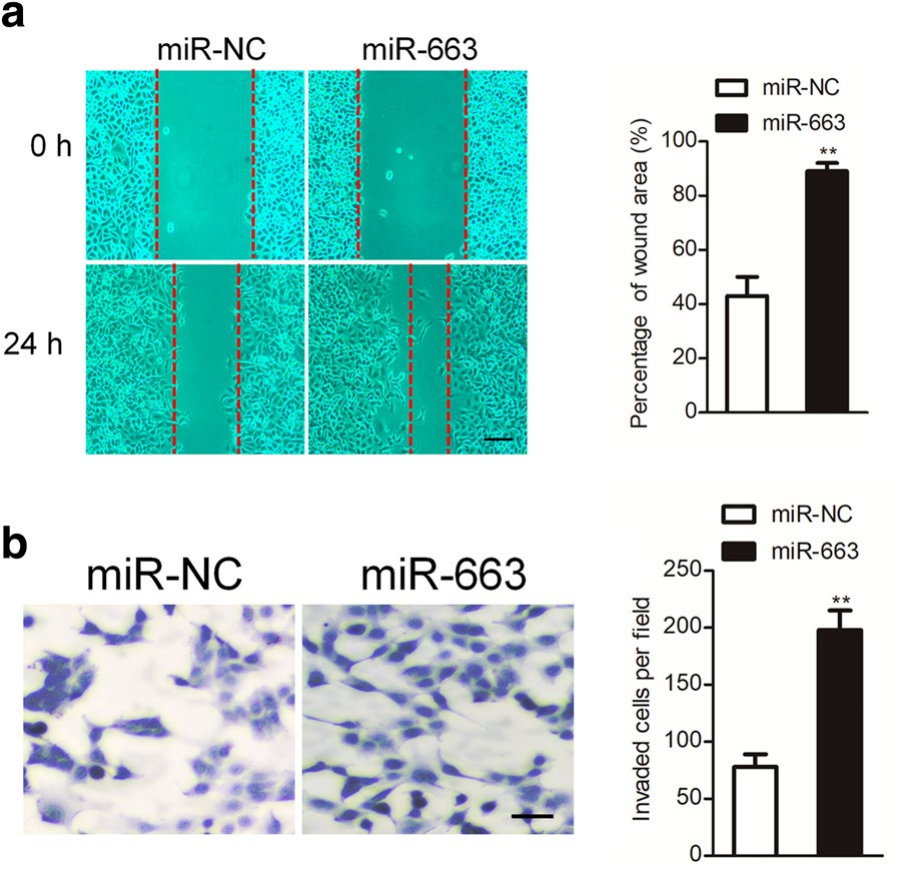
MiR-663 over-expression promotes SKOV3 cells mobility and invasive. **a** Wound closure analysis shown the migration of miR-NC transfected cells and miR-663 over-expressing SKOV3 cells. **b** Transwell invasion analysis shown the invasive of miR-NC transfected cells and miR-663 over-expressing SKOV3 cells. N = 3, **P < 0.01, compared to miR-NC

Given the correlation of cancer cell migration and distant metastasis in ovarian cancer, we further detected the biology effects of miR-663 on the migration and invasion of ovarian cancer cell. As shown in Fig. 3a, the wound healing assays suggested that miR-663 over-expression significantly increased the wound closure of SKOV3 cell in vitro. Consistently, over-expression of miR-663 promoted the invasion of SKOV3 cell compared to control cell (Fig. 3b).

### TUSC2 is negative regulated bymiR-663 in SKOV3 cell

**Fig. 4:**
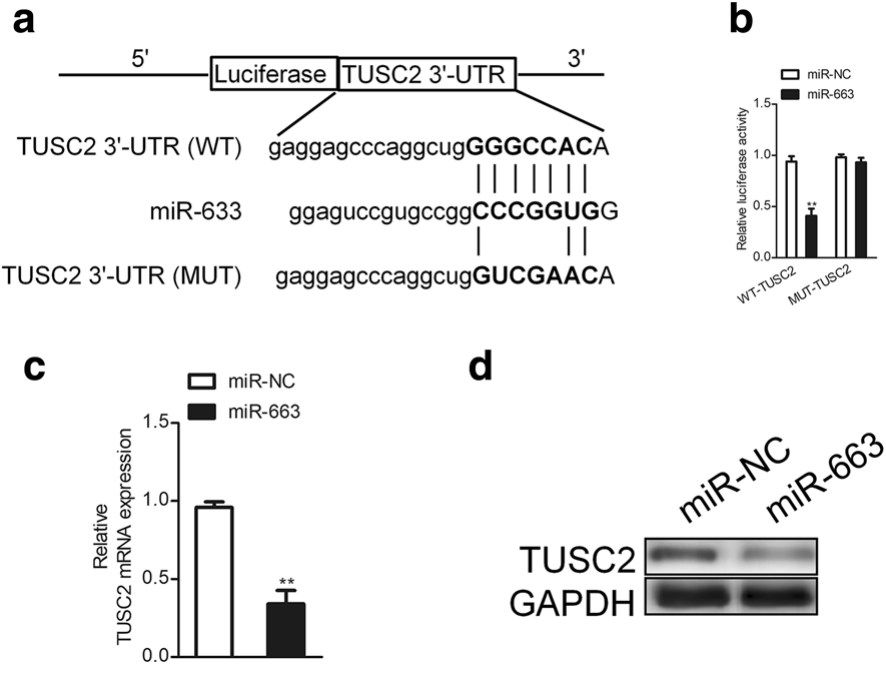
TUSC2 is the target of miR-663 in SKOV3 cell. **a** Sequence alignment of wild-type and mutant type of miR-663-binding sites in 3′-UTR of TUSC2. **b** Luciferase activity was reduced in SKOV3 that co-transfected miR-663 along with WT TUSC2-3′-UTR. **c** The mRNA level of TUSC2 in miR-663 over-expressing SKOV3 was detected by qRT-PCR method. **d** Immunoblotting analysis of TUSC2 expression in miR-663 over-expressing SKOV3 cells. N = 3, **P < 0.01, compared to miR-NC

Then, the targets of miR-663 were predicted using online analysis tools, including TargetScan (http://www.targetscan.org), miRTarBase (http://mirtarbase.mbc.nctu.edu.tw/php/index.php) and miRDB (http://www.mirdb.org/). Total six common target genes were obtained from three bioinformatics analysis tools (Additional file 1: Figure S1A). In order to identify the direct gene of miR-663 the mRNA levels of these genes in miR-663 or miR-NC transfected SKOV3 cell were detected using qRT-PCR assay. As shown in Additional file 1: Figure S1B, the mRNA level of TUSC2 was significantly inhibited by miR-663 in SKOV3 cell. The complementary sequences of miR-663 were discovered in 3′-UTR of TUSC2 mRNA (Fig. 4a). Then, we conducted the luciferase analysis using miR-663 over-expressing SKOV3 cells that were transfected with wild type (WT) or mutant type (MUT) of TUSC2-3′-UTR. As shown in Fig. 4b, the luciferase activity of cells that were cotransfected with miR-663 and WT-TUSC2-3′-UTR was reduced whereas the luciferase activity was not affected in cells that were cotransfected with MUT TUSC2-3′-UTR and miR-663. Consistently, up-regulation of miR-663 inhibited the mRNA and protein level of TUSC2 in ovarian carcinoma SKOV3 cell (Fig. 4c, d). Evidence to date indicates that TUSC2, also known as FUS1, behaves as a tumor suppressor in several types of cancers, including lung cancer, breast cancer and thyroid cancer [21, 22]. Previous investigations have indicated that ectopic expression of TUSC2 inhibited cell proliferation, survival, migration, and invasion, and increased tumor cell death [23]. Science the expression of TUSC2 was negatively regulated by miR-663, we speculated that miR-663 promoted the growth, migration and invasion of SKOV3 via inhibiting the level of TUSC2.

### The effect of miR-663 on SKOV3 cell was inhibited by over-expression of TUSC2

**Fig. 5:**
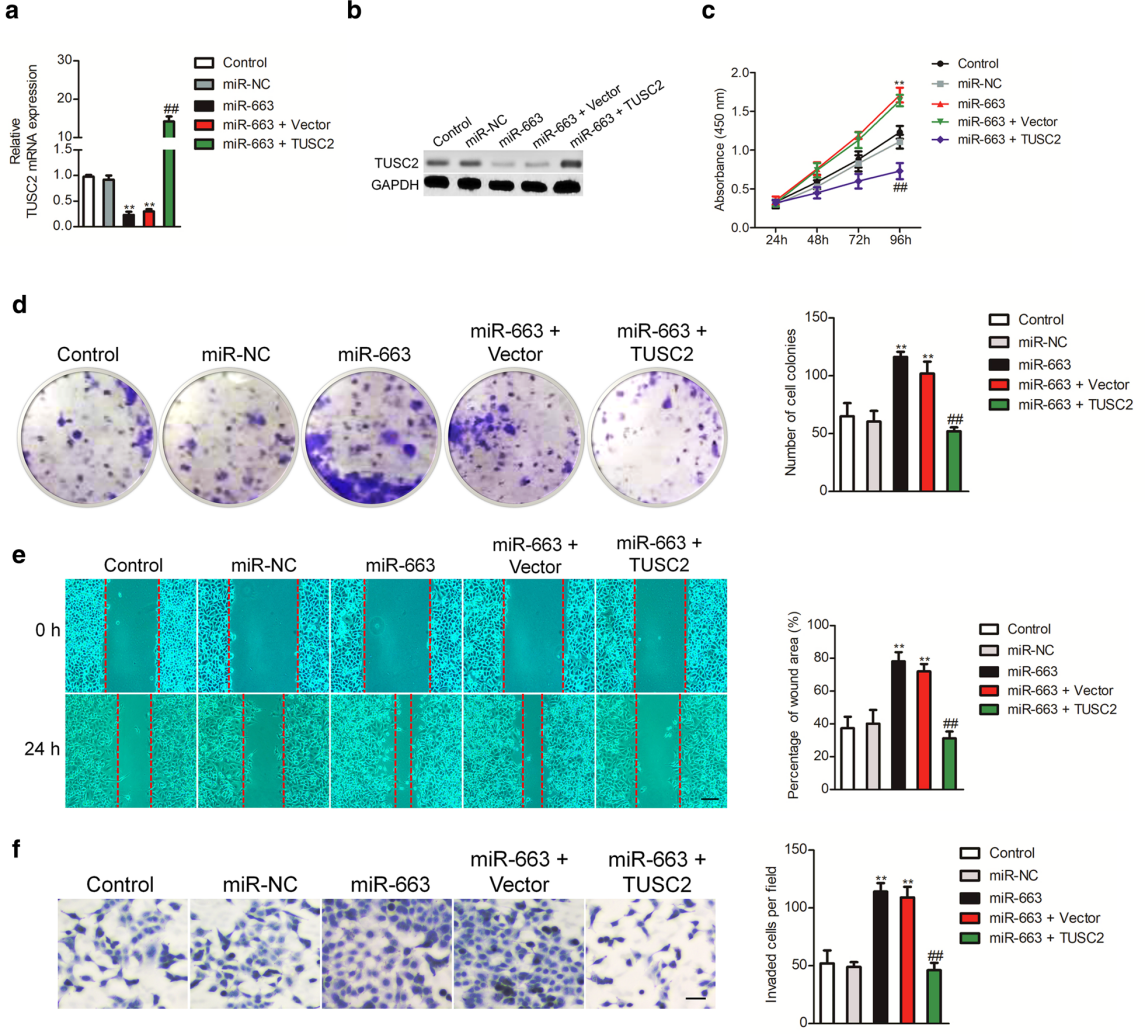
TUSC2 is involves in the effect of miR-663 in SKOV3 growth and mobility. **a** The qRT-PCR assay of TUSC2 in SKOV3 cells that co-transfected with TUSC2 over-expression plasmid along with either miR-NC or miR-663. **b** SKOV3 cells were co-transfected with miR-663 and TUSC2 over-expressing plasmid and the level of TUSC2 was assessed by western blot. **c** CCK-8 assay shown in the proliferation of SKOV3 cell that co-transfected with miR-663 and TUSC2 over-expression plasmid. **d** Colony formation analysis of SKOV3 cells co-transfected with miR-663 and TUSC2 over-expressing plasmid. **e** Wound closure analysis was performed to measure the migration of indicated SKOV3 cells. **f** Transwell assay was conducted to assess the invasiveness of indicated SKOV3 cells. N = 3, **P < 0.01, compared to miR-NC, ^##^*P* < 0.01 in comparison with miR-663 + TUSC2

To future investigate the actions of TUSC2 in miR-663 mediated SKOV3 growth, migration and invasion, SKOV3 ovarian carcinoma cells were co-transfected with pCDNA3.1-TUSC2 and miR-663 mimics. The level of TUSC2 in SKOV3 cells was restored in SKOV3 cells that were cotransfected with TUSC2 over-expressing plasmid and miR-663 mimics (Fig. 5a, b). As expected, over-expression of TUSC2 significantly suppressed the SKOV3 cell growth, colony formation, migration and invasion that were induced by miR-663 (Fig. 5c–f). These findings reveal that miR-663 promotes ovarian cancer SKOV3 cell growth and metastasis by inhibiting TUSC2.

### TUSC2 is negatively correlated with miR-663 in ovarian cancer tissue

**Fig. 6:**
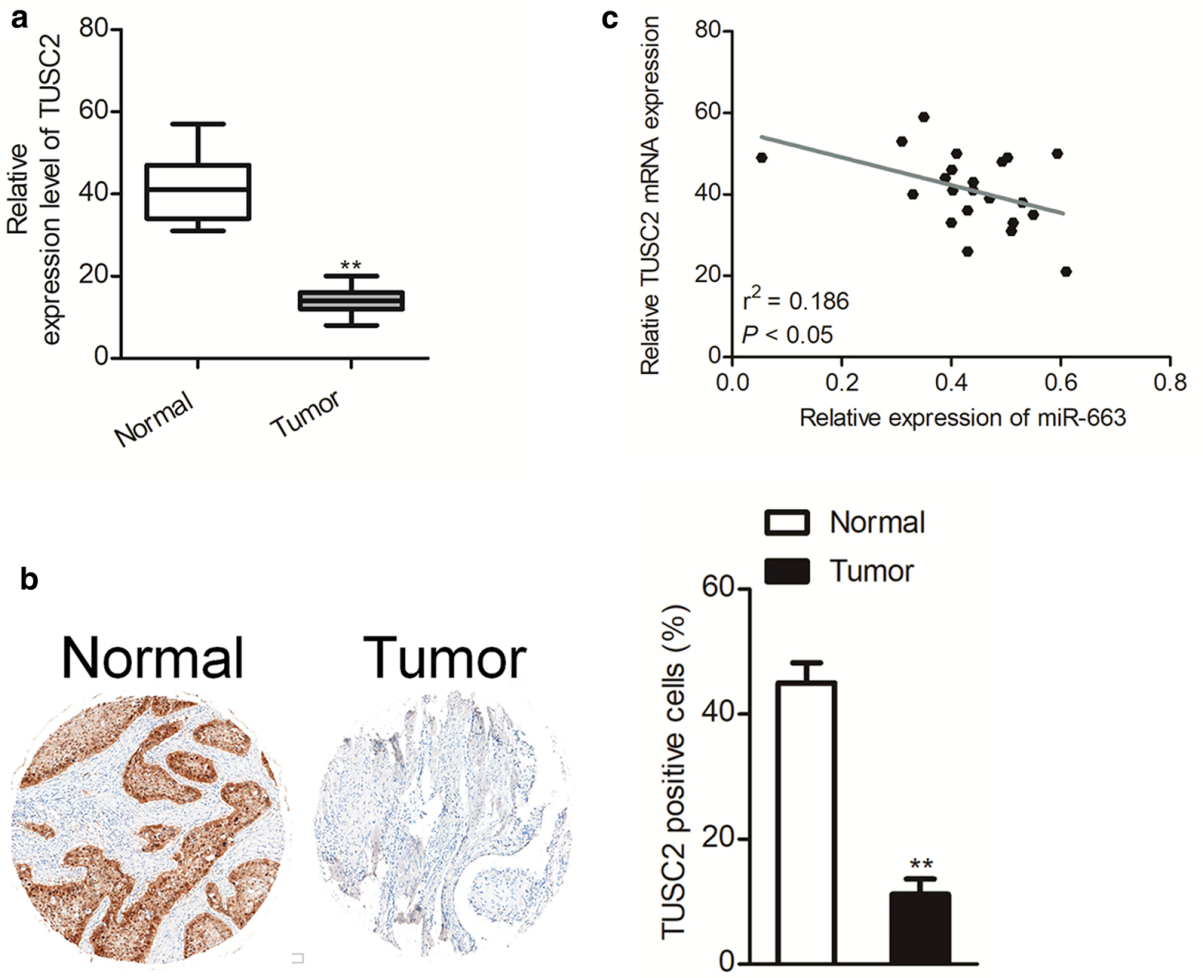
TUSC2 is negatively associated with miR-663 in ovarian carcinoma. **a** The level of TUSC2 in 23 paired ovarian carcinoma and adjacent normal samples was detected by qRT-PCR. **b** Immunohistochemical staining of TUSC2 in ovarian carcinoma tissue samples and corresponding normal. N = 3. **P < 0.01, compared to normal. **c** Spearman’s correlation analysis of the relationship of TUSC2 and miR-663 in ovarian carcinoma (n = 23)

Finally, we explored the level of TUSC2 in 23 pairs of ovarian carcinoma and matched normal tissues by qRT-PCR assay. As shown in Fig. 6a, the levels of TUSC2 were lower in the ovarian cancer tissues in comparison with the adjacent normal tissues. Immunohistochemical staining analysis future proved that TUSC2 was down-regulated in ovarian cancer tissue compared to the matched normal tissue (Fig. 6b). Furthermore, the Spearman’s correlation analysis showed that TUSC2 was negatively associated with miR-663 level in ovarian cancer (Fig. 6c). Altogether, these data demonstrate that TUSC2 is down-expressed and negatively associated with miR-663 in ovarian cancer tissue.

## Discussion

MicroRNAs play vital roles in tumorigenesis and the progression of diverse cancers [24, 25]. MicroRNAs can regulate the expression of tumor related genes, and act as a tumor suppressor gene or an oncogene, playing an important role in the diagnosis and treatment of cancer [26]. In the current study, we explored the precise roles of miR-663 in ovarian cancer cell growth, migration and invasion. We found that miR-663 was up-regulated in ovarian carcinoma tissues and cell lines when compared to normal specimens and ovarian surface epithelial cell, respectively. Furthermore, over-expression of miR-663 promoted the growth, colony formation, migration and invasion of ovarian carcinoma SKOV3 cell in vitro. Consistently, the up-regulation of miR-663 increased the tumor growth of SKOV3 cell in vivo. Furthermore, we demonstrated that miR-663 facilitated ovarian cancer cell growth and progression by suppressing TUSC2.

Recently, there’s increasing study and focus on the roles of miR-663 in the metastasis, chemotherapy-resistance and tumor-associated angiogenesis of various cancers. For example, miR-663 plays vital roles in nasopharyngeal carcinoma, pancreatic cancer and acute myeloid leukemia (AML) [27]. MiR-663 promotes nasopharyngeal carcinoma cells growth by means of directly targeting cyclin-dependent kinase inhibitor 2A (CDKN2A) and sustains non-small cell lung cancer (NSCLC) via restraining mitochondrial outer membrane permeabilization (MOMP) through p53 up-regulated modulator of apoptosis/Bcl-2 binding component 3 (PUMA/BBC3) and B-cell translocation gene 2 (BTG2). Nevertheless, the expression pattern and roles of miR-663 in ovarian carcinoma is not well investigated. In this study, we analyzed the levels of miR-663 in ovarian carcinoma tissues and demonstrated that up-expression of miR-663 facilitated the growth, migration and invasion of ovarian carcinoma SKOV3 cell. Bioinformatics analysis identified TUSC2 as the directly target of miR-663 and miR-663 over-expression inhibited the level of TUSC2 in ovarian cancer cell. The cancer suppressive functions of TUSC2 have been previously identified in other cancers. TUSC2 sensitizes non-small cell lung cancer cells to AKT inhibitor, MK2206 in LKB1 dependent manner [28]. Over-expression of TUSC2 inhibits the growth, mobility and invasion of glioblastoma cells [18]. In addition, TUSC2 is found to be down-regulated in nasopharyngeal carcinoma and negatively regulates proliferation and cell cycle progression [27]. On account of these observations, we speculated that miR-663 might participate into the malignant phenotype of ovarian carcinoma cells. Firstly, our data suggested that miR-663 was indeed up-regulated in ovarian carcinoma tissues when compared with control normal samples. Moreover, we demonstrated that over-expression of miR-663 remarkably facilitated the growth, mobility and aggressiveness of ovarian carcinoma cells. All these results were consistent with the previous study, in which miR-663b promotes the proliferation, migration and invasion of nasopharyngeal carcinoma cell through targeting TUSC2 [27]. Previous studies demonstrate that miRNAs negatively regulate protein expression through binding to 3′-UTR of target genes. In our data, luciferase reporter analysis suggested that miR-663 directly bind to 3′-UTR of TUSC2. What’s more, restoration of TUSC2 neutralized the promotion of growth and migration in miR-663 transfected ovarian cancer cell. Since miR-663 plays such an important regulatory role in ovarian cancer, if we can develop a drug targeting miR-663 or interfering the interaction between miR-663 and downstream target gene, it may be able to provide a new therapeutic approach for the treatment of ovarian cancer. Of course, these assumptions need more experimental data to validate and more supports from clinical data. Meanwhile, the primary limitation of this work is that we only analyzed fewer ovarian cancer clinical tissues and cell lines. Hence, more precise investigations are needed for profoundly explore the latent therapeutic role of miR-663 in ovarian carcinoma.

## Conclusion

In conclusion, miR-663 promoted ovarian cancer cell growth and progression by targeting TUSC2. Furthermore, these results provided insight into the potential therapeutic value of miR-663 in reducing ovarian cancer cell growth and aggressiveness.

## Additional file

**Additional file 1: Figure S1.** TUSC2 is the target of miR-663 in SKOV3 cell. **A.** Venn graph represented the number of candidate common target genes determined by three bioinformatics analysis. **B.** SKOV3 cell was transfected with miR-NC or miR-663 and the levels of potential target genes were measured by qRT-PCR assay. **P < 0.01 in comparison to control.

## Abbreviations

TUSC2: tumor suppressor candidate 2
3′-UTR: 3′-untranslated region
qRT-PCR: quantitative real-time PCR
GAPDH: glyceraldehyde-3-phosphate dehydrogenase
miR-NC: miR-negative control
DMEM: Dulbecco’s modified Eagle medium
WT: wild type
MUT: mutant type
NSCLC: non-small cell lung cancer
MOMP: mitochondrial outer membrane permeabilization
PUMA/BBC3: p53 up-regulated modulator of apoptosis/Bcl-2 binding component 3
BTG2: B cell translocation gene 2
