# Supplementary material for: MicroRNA-663 facilitates the growth, migration and invasion of ovarian cancer cell by inhibiting TUSC2
Source: Biol Res. 2019 Apr 3;52:18. doi: 10.1186/s40659-019-0219-6 (PMC6448305; doi:10.1186/s40659-019-0219-6)
Supplement: Supplementary file 1 — Additional file 1: Figure S1. TUSC2 is the target of miR-663 in SKOV3 cell. A. Venn graph represented the number of candidate common target genes determined by three bioinformatics analysis. B. SKOV3 cell was transfected with miR-NC or miR-663 and the levels of potential target genes were measured by qRT-PCR assay. **P < 0.01 in comparison to control. [file 40659_2019_219_MOESM1_ESM.docx]

**
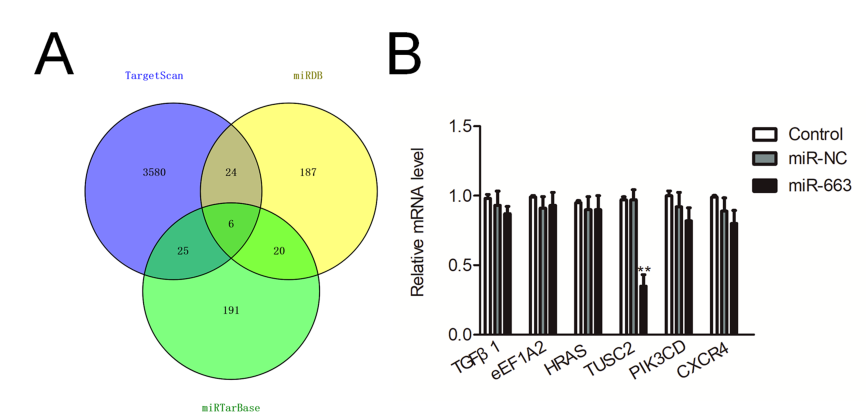
**

**Additional file 1: Figure 1.** TUSC2 is the target of miR-663 in SKOV3 cell. **A.** Venn graph represented the number of candidate common target genes determined by three bioinformatics analysis. **B**. SKOV3 cell was transfected with miR-NC or miR-663 and the levels of potential target genes were measured by qRT-PCR assay. ^**^*P* < 0.01 in comparison to control.
